# Analysis of motor, cognitive and language performance of infants undergoing treatment for congenital hypothyroidism

**DOI:** 10.1016/j.jped.2024.08.008

**Published:** 2024-10-11

**Authors:** Môyra A. Romero, Maura M.F. Goto, Michelle P.C. d'Ouro, Maria Cecília M.P. Lima, Vivian F. Dutra, Carolina T. Mendes-dos-Santos, Denise C.C. Santos

**Affiliations:** a Universidade Metodista de Piracicaba (UNIMEP), Programa de Pós-graduação em Ciências do Movimento Humano, Piracicaba, SP, Brazil; b Universidade Estadual de Campinas (UNICAMP), Faculdade de Ciências Médicas, Departamento de Pediatria, Campinas, SP, Brazil; c Universidade Estadual de Campinas (UNICAMP), Faculdade de Ciências Médicas, Programa de Pós-graduação em Saúde da Criança e do Adolescente, Campinas, SP, Brazil; d Universidade Estadual de Campinas (UNICAMP), Faculdade de Ciências Médicas, Departamento de Desenvolvimento Humano e Reabilitação, Campinas, SP, Brazil; e Universidade Estadual de Campinas (UNICAMP), Faculdade de Ciências Médicas, Programa de Triagem Neonatal da UNICAMP/CIPOI, Campinas, SP, Brazil

**Keywords:** Congenital hypothyroidism, Neonatal screening, Child development

## Abstract

**Objective:**

Investigate the association between the age of treatment onset and confirmatory TSH level (as an indicator of severity) with a greater risk of developmental delay in infants with congenital hypothyroidism (CH).

**Method:**

The authors conducted a cross-sectional, observational, unmatched case-control study at a Brazilian neonatal screening reference center. Seventy-seven infants with CH (mean age: 12 ± 6.4 months) were examined. The authors evaluated their performance using the Bayley-III Screening Test and categorized them as “LOWER RISK” (competent category) or “GREATER RISK” (combined at-risk + emergent categories) for developmental delay based on the 25^th^ percentile cutoff.

**Results:**

Infants with CH are at a higher risk of non-competent performance in cognition, receptive language, fine motor skills, and gross motor skills when compared to infants without CH. This risk is more pronounced in infants with more severe indications of CH (TSH > 30 μUI/L in the confirmatory test) for cognition (OR = 5.64; *p* = 0.01), receptive language (OR = 14.68; *p* = 0.000), fine motor skills (OR = 8.25; *p* = 0.000), and gross motor skills (OR = 5.00; *p* = 0.011).

**Conclusion:**

The level of TSH in the confirmatory test can be a good indicator for identifying infants with CH who are at a higher risk of non-competent performance in cognition, receptive language, and motor skills. Monitoring development, early detection of delays, and intervention programs are particularly important for infants with CH.

## Introduction

Congenital hypothyroidism (CH) is a condition characterized by an underactive or absent thyroid gland in infants. The thyroid gland is responsible for producing essential hormones for normal growth and development. CH stands as the most frequent congenital endocrine disorder. The American Academy of Pediatrics reported that CH affected about one in every 3,000 to 4,000 newborn infants in 2006.^1^ Recently, there was reported an incidence rate of 1:1,030 to 1:2,679 live births.^2^ Early diagnosis and treatment of CH are crucial to prevent developmental delays, intellectual disabilities, and other potential complications.

Clinical manifestations of CH in neonates are generally absent, making neonatal screening (heel prick test) vital for the early identification and treatment of the condition.^3^ Neonatal screening programs, introduced over the last 50 years in most industrialized countries, with adequate treatment, have liberated children from severe mental impairment. However, developmental outcomes of treated CH individuals have shown mild psychomotor impairment, persisting from infancy to adult life.

Compared to individuals without CH, several studies have identified suboptimal psychomotor development in infancy, adolescence and extending into adulthood in the presence of CH.^4^, ^5^, ^6^, ^7^, ^8^, ^9^, ^10^, ^11^ In addition to the timing and severity of thyroid hormone deficiency, developmental outcomes can also be influenced by the time of diagnosis, age of treatment onset, dose of thyroid hormone replacement, age of thyroid hormone normalization and family adherence to treatment. Multiple factors influencing the developmental outcomes of children affected by CH potentially account for its vulnerability and the differing outcomes observed in various studies.^1^^,^^4^^,^^6^^,^^8^^,^^11^, ^12^, ^13^, ^14^, ^15^, ^16^

The purpose of neonatal screening for CH is to prevent, through early detection and treatment, neurodevelopmental disability, including severe mental retardation and to optimize developmental outcomes.^1^^,^^7^^,^^17^ Despite the success of screening programs in preventing severe disabilities, numerous studies indicate some degree of dysfunction in various areas of development, even in treated individuals.^5^^,^^7^, ^8^^,^^18^, ^19^, ^20^, ^21^, ^22^, ^23^

Although monitoring neuropsychomotor development is recommended by the American Academy of Pediatrics^24^ and the European Society for Pediatric Endocrinology,^17^ it is not included in the services offered by neonatal screening programs.^1^^,^^25^, ^26^ The present study focuses on the age of treatment onset and the confirmatory TSH (thyroid-stimulating hormone) level as an indicator of severity to investigate their association with a greater risk of developmental delay. This research was motivated by the risk that CH poses to infant development and, consequently, the need for monitoring this development to be included in neonatal screening services.

Thus, the research questions (RQ) that this study aimed to answer were:

RQ1: Are infants under treatment for CH at a greater risk for developmental delays in motor, cognitive and language domains compared to healthy infants?
RQ2: Are infants with CH, treated within the neonatal period (up to 28 days) or after the neonatal period, at a greater risk for developmental delay in the areas of motor, language, and cognition compared to healthy infants?
RQ3: Are infants with CH with confirmatory TSH levels up to 30 μUI/L or above 30 μUI/L at a greater risk for developmental delay in the areas of motor, language and cognition compared to healthy infants?

## Methods

A cross-sectional, observational, unmatched case-control study was conducted at a Brazilian neonatal screening reference center situated in São Paulo State. The local ethics committee approved the study (#1008-2011) and parents or guardians provided informed consent.

### Participants

Seventy-seven infants with CH, with a mean age of 12 ± 6.4 months, were examined. They were followed up at the neonatal screening reference center. The CH group comprised infants under confirmed CH treatment. The control group included 49 disease-free infants (mean age: 13 ± 4.6 months) attending daycare. All participants had a birth weight > 2,500 g and were free from birth or neonatal complications and diseases, except for hypothyroidism in the CH group. The exclusion criteria included developmental delays or genetic syndromes, enrollment in a rehabilitation program, birth defects, or any other health condition that could affect child development.

To address the research questions, participants were categorized by the presence of congenital hypothyroidism, the age of treatment onset, and the serum confirmatory TSH levels. The studied groups were composed based on the following research questions (RQ):

***RQ1*** - Are infants under treatment for congenital hypothyroidism at greater risk for developmental delays in motor, cognitive and language domains compared to healthy infants?
  - ***CHG:*** Infants with CH (n = 77)
  - ***CG:*** Control group, infants without CH (n = 49)
***RQ2*** - Are infants with congenital hypothyroidism, treated within the neonatal period (up to 28 days) or after that period, at a greater risk for developmental delay in motor, language and cognition compared to healthy infants?
  - ***CHG ≤ 28 DAYS:*** Infants with CH treated within the neonatal period (n = 46)
  - ***CHG > 28 DAYS:*** Infants with CH treated after 28 days of age (n = 31)
  - ***CG:*** Control group, infants without CH (n = 49)
***RQ3*** - Are infants with congenital hypothyroidism, with confirmatory TSH levels up to 30 μUI/L or above, at greater risk for developmental delay in motor, language and cognition compared to healthy infants?
  - ***TSH ≤ 30:*** Infants with CH and TSH levels up to 30 μUI/L (n = 51)
  - ***TSH > 30:*** Infants with CH and TSH levels above 30 μUI/L (n = 26)
  - ***CG:*** Control group, infants without CH (n = 49)

### Instrument

The Bayley Scales of Infant and Toddler Development Screening Test, Third Edition (Bayley-III Screening Test) was employed to assess motor (fine and gross), cognitive and language (receptive and expressive) development.^27^ The Bayley-III Screening Test is recognized as an effective tool for identifying infants eligible for early intervention.

The Bayley-III Screening Test identifies the degree of risk for developmental delay in children up to 42 months. For each subtest, the child's performance is interpreted based on a Gaussian distribution such as:

- ***COMPETENT (low risk for developmental delay):*** child's performance at or above the 25^th^ percentile or −0.67 SD;
- ***EMERGENT (some risk for developmental delay):*** child's performance below the 25th percentile or −0.67 SD;
- ***AT RISK (at risk for developmental delay):*** child's performance below the 2nd percentile or −2.0 SD.

In this study, the infant's performance was dichotomized using the 25th percentile cutoff as ***LOWER RISK*** (competent category) or ***GREATER RISK*** (combined at-risk + emergent categories) for developmental delay. Experienced examiners from an interdisciplinary research group (including a pediatrician, physiotherapists, speech pathologists and psychologist) conducted the assessments. The assessment team was blind to the infant's neonatal and screening/treatment data.

### Statistical analysis

Chi-square tests (χ^2^) with a degree of freedom = 1 and odds ratio (OR) with a 95% confidence interval (CI) were used for the association analysis. The risk association was considered significant when the CI lower limit was greater than one. Group characteristics were compared using descriptive statistics and independent Student's t-test or chi-square test. A statistical significance level was set at alpha = 0.05.

## Results

The study comprised a total of 126 infants: 77 with CH and 49 infants without CH (controls). The groups exhibited homogeneity regarding sex (*p* = 0.238), gestational age (*p* = 0.755), birth weight (*p* = 0.280) and age at the time of infant performance evaluation (*p* = 0.274). Table 1 shows the main characteristics of each studied group.

**Table 1:** Characterization of the studied groups.

Characteristics	CHG (n = 77)	CG (n = 49)	*p* value
Female	28 (36.4%)	23 (46.9%)	0.238^a^
Male	49 (63.6%)	26 (53.1%)	
Gestational age (wk)	39 ± 1.3	39 ± 1.4	0.755^b^
Birth weight (in grams)	3.292 ± 401	3.372 ± 403	0.280^b^
Assessment age (months)	11 ± 6	12 ± 5	0.274^b^

a Chi-square test.

b Parametric t-test.

Results presented a significant association between the age of treatment initiation and an indicator of CH severity, the confirmatory TSH (X^2^(1) = 7.218; *p* = 0.007). It was found that the group treated earlier (CHG ≤ 28 days) consisted of 80.8% of infants exhibiting higher TSH levels, indicating more severe HC.

Table 2 shows that in response to the first research question, infants under CH treatment, regardless of the age at treatment onset or serum confirmatory TSH levels, were at a greater risk for developmental delay in fine motor (OR = 4.79; *p* = 0.004), gross motor (OR = 4.21; *p* = 0.009) and receptive language (OR = 8.81; *p* = 0.001) compared to healthy peers.

**Table 2:** Comparison of the domains of cognition, language, and motor skills between the CH affected groups (CHG) against the control group (CG), considering n subjects, with odds ratio (OR), confidence interval (IC) and chi-square testing (χ^2^).

Domain	Group	n	Frequency		OR	95% CI	χ^2^^a^
			Greater risk	Lower risk			
Cognition	CHG	77	12 (15.6%)	65 (84.4%)	2.83	0.75–10.6	2.55 (0.110)
	CG	49	3 (6.1%)	46 (93.9%)	-	-	-
Receptive language	CHG	77	21 (27.3%)	56 (72.7%)	**8.81**	**1.96–39.5**	**10.8 (0.001)**
	CG	49	2 (4.1%)	47 (95.9%)	-	-	-
Expressive language	CHG	77	34 (44.2%)	43 (55.8%)	1.36	0.65–2.83	0.68 (0.400)
	CG	49	18 (36.7%)	31(63.3%)	-	-	-
Fine motor	CHG	77	23 (29.9%)	54 (70.1%)	**4.79**	**1.54–14.9**	**8.38 (0.004)**
	CG	49	4 (8.2%)	45 (91.8%)	-	-	-
Gross motor	CHG	77	21 (27.3%)	56 (72.7%)	**4.21**	**1.35–13.2**	**6.87 (0.009)**
	CG	49	4 (8.2%)	45 (91.8%)	-	-	-

Significant associations are shown in bold.

a Chi-square tests for degree of freedom = 1, values between brackets are *p*-values.

Regarding the second research question, infants treated for CH within the neonatal period were at a higher risk for cognitive developmental delay (OR = 4.81; *p* = 0.01) compared to healthy peers. For fine motor skills (CHG ≤ 28 days: OR = 4.92 and *p* = 0.005; CHG > 28 days: OR = 4.60 and *p* = 0.013), gross motor skills (CHG ≤ 28 days: OR = 3.53 and *p* = 0.035; CHG > 28 days: OR = 5.35 and *p* = 0.005) and receptive language (CHG ≤ 28 days: OR = 8.29 and p = 0.002; CHG > 28 days: OR = 9.61 and *p* = 0.001), infants with CH were at greater risk for developmental delay in both groups (treatment initiated within or after the neonatal period), displaying similar risk regardless of the age of treatment onset (Table 3).

**Table 3:** Comparison of the domains of cognition, language, and motor skills between the CH-affected groups which initiated treatment before the neonatal period (CHG ≤ 28), after the neonatal period (CHG > 28) against the control groups (CG), considering n subjects, with odds ratio (OR), confidence interval (IC) and chi-square testing (χ^2^).

Domain	Group	n	Frequency		OR	95% CI	χ^2^^a^
			Greater risk	Lower risk			
Cognition	CHG ≤ 28	46	11 (23.9%)	35 (76.1%)	**4.81**	**1.24–18.6**	**5.97 (0.010)**
	CHG > 28	31	1 (3.2%)	30 (96.8%)	0.51	0.05–5.14	0.33 (0.560)
	CG	49	3 (6.1%)	46 (93.9%)	-	-	-
Receptive language	CHG ≤ 28	46	12 (26.1%)	34 (73.9%)	**8.29**	**1.74–39.5**	**9.14 (0.002)**
	CHG > 28	31	9 (29.0%)	22 (71.0%)	**9.61**	**1.91–48.3**	**9.96 (0.001)**
	CG	49	2 (4.2%)	47 (95.9%)	-	-	-
Expressive language	CHG ≤ 28	46	23 (50.0%)	23 (50.0%)	1.72	0.75–3.90	1.70 (0.190)
	CHG > 28	31	11 (35.5%)	20 (64.5%)	0.94	0.37–2.41	0.01 (0.900)
	CG	49	18 (36.7%)	31 (63.2%)	-	-	-
Fine motor	CHG ≤ 28	46	14 (30.4%)	32 (69.6%)	**4.92**	**1.48–16.3**	**7.66 (0.005)**
	CHG > 28	31	9 (29.0%)	22 (71.0%)	**4.60**	**1.27–16.6**	**6.07 (0.013)**
	CG	49	4 (8.2%)	45 (91.8%)	-	-	-
Gross motor	CHG ≤ 28	46	11 (23.9%)	35 (76.1%)	**3.53**	**1.03–12.1**	**4.42 (0.035)**
	CHG > 28	31	10 (32.3%)	21 (67.7%)	**5.35**	**1.50–19.1**	**7.63 (0.005)**
	CG	49	4 (8.2%)	45 (91.8%)	-	-	-

Significant associations are shown in bold.

a Chi-square tests for degree of freedom = 1, values between brackets are *p*-values.

Table 4 illustrates that infants with higher serum confirmatory TSH were at a greater risk for cognitive developmental delay compared to control peers (OR = 5.64; *p* = 0.01). In the domains of fine motor, gross motor and receptive language, infants with CH were at a greater risk for developmental delay in both groups (higher or lower serum TSH). However, the risk was notably higher among those exhibiting more severe CH (confirmatory TSH > 30 μUI/L) for cognition (OR = 5.64; *p* = 0.01), receptive language (OR = 14.68; *p* = 0.000), fine motor skills (OR = 8.25; *p* = 0.000) and gross motor skills (OR = 5.00; *p* = 0.011).

**Table 4:** Comparison of the domains of cognition, language, and motor skills between the CH-affected groups exhibiting confirmatory TSH ≤ 30 μUI/L and those with TSH > 30 μUI/L against the control groups (CG), considering n subjects, with odds ratio (OR), confidence interval (IC) and chi-square testing (χ^2^).

Domain	Group	n	Frequency		OR	95% CI	χ^2^^a^
			Greater risk	Lower risk			
Cognition	TSH > 30	26	7 (26.9%)	19 (73.1%)	**5.64**	**1.31–24.2**	6.36 (0.010)
	TSH ≤ 30	51	5 (9.8%)	46 (90.2%)	1.66	0.37–7.38	0.46 (0.490)
	GC	49	3 (6.1%)	46 (93.9%)	-	-	-
Receptive language	TSH > 30	26	10 (38.5%)	16 (61.5%)	**14.7**	**2.90–74.3**	**14.9 (0.000)**
	TSH ≤ 30	51	11 (21.6%)	40 (78.4%)	**6.46**	**1.35–30.9**	**6.75 (0.009)**
	GC	49	2 (4.1%)	47 (95.9%)	-	-	-
Expressive language	TSH > 30	26	15 (57.7%)	11 (42.3%)	2.34	0.88–6.20	3.02 (0.080)
	TSH ≤ 30	51	19 (37.2%)	32 (62.8%)	1.02	0.45–2.30	0.00 (0.950)
	GC	49	18 (36.7%)	31 (63.3%)	-	-	-
Fine motor	TSH > 30	26	11 (42.3%)	15 (57.7%)	**8.25**	**2.28–29.8**	**12.4 (0.000)**
	TSH ≤ 30	51	12 (23.5%)	39 (76.5%)	**3.46**	**1.03–11.1**	**4.39 (0.036)**
	GC	49	4 (8.2%)	45 (91.8%)	-	-	-
Gross motor	TSH > 30	26	8 (30.8%)	18 (69.2%)	**5.00**	**1.33–18.7**	**6.45 (0.011)**
	TSH ≤ 30	51	13 (25.5%)	38 (74.5%)	**3.84**	**1.15–12.8**	**5.31 (0.020)**
	GC	49	4 (8.2%)	45 (91.8%)	-	-	-

Significant associations are shown in bold.

a Chi-square tests for degree of freedom = 1, values between brackets are *p*-values.

## Discussion

In the present study of 77 infants with CH, the authors have demonstrated that a significantly larger proportion of the CH population experienced impairments in cognition, receptive language, and motor skills compared to the general population. The increased risk of developmental issues became more evident when the groups were divided based on the level of serum confirmatory TSH.

The results concerning CHG, irrespective of the age at the onset of treatment or the confirmatory TSH level, indicated that CHG had a higher likelihood of exhibiting inadequate performance in receptive language and motor skills. This suggests that the congenital condition affects infant development, even when these infants are under the care of a reference service with good screening and treatment indicators.

The authors can infer that this occurs due to insufficient thyroid hormones during the prenatal phase of neurological development. Prenatal hormonal insufficiency is associated with deficits extending into childhood, including limitations in language, fine motor skills, auditory processing, attention and memory.^28^ Thyroid hormones (TH) play an important role in the development of the Central Nervous System (CNS) spanning from the fetal to the postnatal period. Thus, the timing and severity of the TH deficiency influence the process of neurogenesis, dendritic proliferation, synapse formation, and myelination.^14^^,^^17^^,^^28^^,^^29^

When the authors analyzed the CH group based on the age at the onset of treatment, we observed that the GREATER RISK for development delay lay in the cognitive domain for the group that initiated treatment before the neonatal period. This prompted us to examine TSH levels (information related to disease severity) because we believed that if treatment commenced earlier, there should be less impact on the individual's development.

The age correlation between treatment initiation and developmental alterations in children, specifically in motor skills and cognition, was already established in previous studies. These studies also found a better correlation with disease severity rather than treatment onset.^7^^,^^8^

In the present findings, the authors discovered that the association of GREATER RISK for development delay based on the age of treatment initiation was also directly related to the severity of CH since 80.8% of infants with TSH levels greater than 30 µUI/L comprised the group of infants with CH younger than 28 days old. These TSH levels supposedly favor neonatal screening because these infants are identified earlier and initiate treatment within what is considered the neonatal period.

A study involving 131 seven-year-old children, found that prenatal factors related to the severity of CH begin to lose their negative influence at the age of seven and disappear by age 12, giving way to postnatal factors, among which the treatment initiation date plays a crucial role.^4^

Studies over time have consistently demonstrated the influence of disease severity on the development of children with CH. Some authors attribute the severity of CH to hormonal insufficiency at the beginning of gestation, leading to neurological impairment during central nervous system development, resulting in deficits despite early hormone replacement.^1^^,^^6^^,^^7^^,^^11^^,^^17^^,^^19^

Regarding the influence of CH severity on infant development, this study indicates that CHG with a TSH level higher than 30 µUI/L in the confirmatory test presented a GREATER RISK for development delay in cognition, receptive language, and motor skills when compared to the control group. This is clinically significant as it provides an early indicator that a subgroup of infants with CH requires increased attention in developmental monitoring and perhaps early developmental stimulation. Individuals with severe CH benefit from early treatment, emphasizing the importance of neonatal screening and hormonal replacement in the early days of life.^1^^,^^4^^,^^7^

Considering the evidence that infants with CH are at a greater risk of developmental alterations, the American Academy of Pediatrics^1^ and the European Society for Pediatric Endocrinology^17^ recommend monitoring neuropsychomotor development, language, and academic performance in children with CH. In Brazil, the Ministry of Health protocol outlines the importance of assessing the neuro-psychomotor development of patients undergoing treatment for hypothyroidism. However, not all neonatal screening services monitor infant development. Nevertheless, the findings of this research underscore the need for monitoring child development within the neonatal screening reference services (SRTN).

The new screening and treatment guidelines for CH from the American Academy of Pediatrics include monthly developmental progress assessments up to 6 months of age, followed by assessments every 2-3 months until 1 year of age, and 3–4-month intervals between 1 and 3 years of age, with semiannual or annual assessment after 3 years.^1^ The European Society for Pediatric Endocrinology suggests specific motor development stimulation and personalized education for children with CH.^25^ Individuals with CH who may have psychomotor disturbances should be monitored by a speech therapist, physiotherapist, and education psychologist.^26^ In addition, when motor issues are present, children with CH should be encouraged to engage in physical activities and be monitored by a physiotherapist.^10^

Overall, the present study demonstrates a GREATER RISK for development delay in cognition, receptive language, and both fine and gross motor skills in infants with CH, especially those with serum TSH levels in the confirmatory test exceeding 30 µUI/L. This underscores the need for increased attention and monitoring of infant development for these infants.

The present study was limited to a cross-sectional assessment of infant performance. However, the authors recognize the importance of a longitudinal study for developmental monitoring, as infant development is dynamic and influenced by various factors (nutritional care, environmental opportunities, parental education level, sanitation), along with medication adherence, particularly in the first three years of life in children with CH. Future research could assess infant development over time longitudinally, tracking cognitive, receptive language, and motor skills development. Additionally, it could explore the influence of the family environment on the development of individuals affected by CH, necessitating educational support for parents, including the provision of opportunities for motor development within the environment.

The findings of the study indicate that infants with CH are at a GREATER RISK of developmental delays in cognition, receptive language, fine motor skills, and gross motor skills when compared to infants without CH. The risk of developmental delay was found to be higher in infants with a higher confirmatory TSH level.

Overall, this study suggests that even infants with CH who are under the care of a specialized service with good screening and treatment indicators are at a higher risk of developmental delays compared to their peers without CH.

In addition to the routine monitoring provided in neonatal screening reference services, monitoring the development to enable early detection of delays and necessary intervention is particularly crucial for infants with CH.

## Financial support

CNPq (process 478770/2012-0) and CAPES/PROSUP.

## Conflicts of interest

The authors declare no conflicts of interest.
